# Decision maker perceptions of resource allocation processes in Canadian health care organizations: a national survey

**DOI:** 10.1186/1472-6963-13-247

**Published:** 2013-07-02

**Authors:** Neale Smith, Craig Mitton, Stirling Bryan, Alan Davidson, Bonnie Urquhart, Jennifer L Gibson, Stuart Peacock, Cam Donaldson

**Affiliations:** 1 Centre for Clinical Epidemiology & Evaluation, Vancouver Coastal Health Research Institute, 7th floor, 828 W 10 Avenue, V5Z1M9, Vancouver, BC, Canada; 2 School of Population and Public Health, University of British Columbia, Vancouver, Canada; 3 Faculty of Health and Social Development, UBC Okanagan, Kelowna, Canada; 4 Northern Health Authority, Prince George, Canada; 5 University of Toronto Joint Centre for Bioethics, Toronto, Canada; 6 Department of Health Policy, Management & Evaluation, University of Toronto, Toronto, Canada; 7 British Columbia Cancer Agency, Vancouver, Canada; 8 Canadian Centre for Applied Research in Cancer Control (ARCC), Vancouver, Canada; 9 Yunus Centre for Social & Business Health, Glasgow Caledonian University, Glasgow, UK

**Keywords:** Resource allocation, Priority setting, Survey research, Canada

## Abstract

**Background:**

Resource allocation is a key challenge for healthcare decision makers. While several case studies of organizational practice exist, there have been few large-scale cross-organization comparisons.

**Methods:**

Between January and April 2011, we conducted an on-line survey of senior decision makers within regional health authorities (and closely equivalent organizations) across all Canadian provinces and territories. We received returns from 92 individual managers, from 60 out of 89 organizations in total. The survey inquired about structures, process features, and behaviours related to organization-wide resource allocation decisions. We focus here on three main aspects: type of process, perceived fairness, and overall rating.

**Results:**

About one-half of respondents indicated that their organization used a formal process for resource allocation, while the others reported that political or historical factors were predominant. Seventy percent (70%) of respondents self-reported that their resource allocation process was fair and just over one-half assessed their process as ‘good’ or ‘very good’. This paper explores these findings in greater detail and assesses them in context of the larger literature.

**Conclusion:**

Data from this large-scale cross-jurisdictional survey helps to illustrate common challenges and areas of positive performance among Canada’s health system leadership teams.

## Background

Resource allocation is a central function of all healthcare delivery systems. We know that priority setting and resource allocation processes need to be both economically sound (making best use of resources to maximize health benefit) and ethical –fair and transparent [1-3]. Evidence from many countries suggests that decision makers struggle to assemble and use relevant evidence [4-7], and to engage clinical stakeholders [8,9] and the public [10-12] in a meaningful fashion. Institutional and cultural barriers stand in the way [13-15].

Our knowledge has accumulated largely through case studies of individual organizations, in Canada [16-22] and elsewhere [23-25]. However cross-jurisdictional, cross-sectional studies of decision makers’ perspectives are relatively few. Some large-scale surveys have been conducted among planning and service delivery agencies within the UK National Health Service; Greener & Powell [26] sought replies from 121 health authorities, while Robinson et al. [27] surveyed 152 Primary Care Trusts. Because primary constitutional authority for organization of healthcare delivery systems in Canada rests with the provincial and territorial jurisdictions, a pan-Canadian survey could potentially be a very rich source of information on political, institutional and cultural factors that influence resource allocation efforts among senior managers with service delivery responsibility.

In the early days of regionalization in Canadian health services, Lomas, Veenstra and Woods surveyed board members, rather than senior executives, about various activities including priority setting and resource allocation [28]. Mitton and Donaldson studied opinions of decision makers in three regional authorities in Alberta [29]; Menon, Stafinski and Martin subsequently engaged senior managers and board members in seven Alberta health regions [30]. Both the latter two studies used semi-structured in-person interviews as their main method. They found that resource allocation is often done on a historical basis, with budgets essentially rolling over from one year to the next. Limited data availability was also mentioned as a barrier to ‘good’ practice. Lack of clinical engagement was also seen as a problem worth addressing.

In this paper, we report on a national survey of senior decision makers in Canadian health organizations. The survey captures decision makers’ views from a much larger number of organizations than previously seen, across different geographies and sizes, including smaller and more rural organizations that have not been typically studied. We anticipate that this paper will be of particular interest to decision-makers in Canada, who may find it useful to see the state of play in other organizations, to identify ways in which they might learn from practices elsewhere, and to generate ideas for leading change efforts in their own organizations. Internationally, senior decision-makers who are committed to the pursuit of excellence in managing health system resources and to achieving ‘high performance’ [31,32] may also learn something of how to operationalize this in practice.

In the following sections, we first outline our survey methodology. Results are presented in four sections. We begin with information about individual respondents and their organizations. We then focus on descriptive findings related to three research questions:

1. What type of resource allocation processes are used by Canadian healthcare organizations? Literature suggests that in Canada and elsewhere, healthcare organizations typically allocate resources on the basis of historical patterns –‘locking in’ to past budget choices [26] -- and/or political rationales [7,33,34], and there are powerful institutional and cultural reasons why this is so [35,36]. However, senior decision makers reportedly desire increased formalization or rationality in priority setting practice [16,20,37-39]. Our aim in the survey was to explore the balance between these different modes in a quantifiable way.

2. Do decision makers in these organizations perceive that these processes are fair? Considerable literature has made the case that priority setting is as much an ethical as an economic or technical activity [1-3]. Some healthcare organizations have made deliberate attempts to incorporate ethical frameworks into their decision making [3,40,41]. Accountability for Reasonableness, or A4R, is a commonly used framework for assessing the actual fairness of resource allocation and priority setting procedures [42-44]. The four original aspects of the A4R model are relevance, publicity, enforcement and appeals. Gibson, Martin & Singer have suggested that Empowerment might be seen as a fifth dimension of A4R [45]; this principle is about allowing opportunity for affected stakeholders to have meaningful input into the process. A number of items based on A4R features were embedded throughout the survey.

3. Overall, how do decision makers rate their current resource allocation practice? This is an attempt to gauge whether or not current practice is seen as successful. Past research in this area is surprisingly limited; as Sibbald et al. note, “only a few studies have presented ideas for evaluating the success of priority setting” [46]; see also [43,47-49]. Given the multi-faceted nature of this concept, the summary measure used here can only be a partial indicator. Nonetheless, we were interested in investigating whether there were individual or organizational factors which influenced respondents’ opinions. We expect to elaborate the concept more fully through subsequent qualitative case study work.

There are, of course, many more survey findings than possibly can be reported in a single article.

In the Discussion and Conclusion, we strive to interpret these findings and indicate what they mean for further study and applied research in the Canadian healthcare system. This survey is one part of a broader project also involving case studies of potentially high performing organizations, whose ultimate aim is to develop a framework and tools to assist healthcare decision makers in assessing their own priority setting practice. Results from subsequent phases will be reported elsewhere.

## Methods

The goal of the online survey was to obtain views of senior decision makers (i.e., those at the vice-president level) in Regional Health Authorities (RHAs) or their closest equivalents, in all 10 Canadian provinces and 3 territories, about their own organization-wide resource allocation processes. The literature was reviewed to identify aspects of priority setting and resource allocation which are thought to be related to effectiveness, success or high performance; this informed the survey content as a whole. Questions were decided by the research team through iterative discussion; detailed discussion of question development is reported elsewhere [50]. The survey was organized in six main sections, with 22 questions in total: descriptive information about the respondents and their organizations (9 questions); an overview of current resource allocation practice (2 questions); organizational values applied to decision making (3 questions); specific factors and criteria considered in decision making (2 questions); organizational culture and context (2 questions); and overall assessment of resource allocation practice (4 questions). The survey also included 2 optional questions meant to set up the case study phase, asking respondents to identify organizations they thought might be considered ‘high performers’ in regard to resource allocation. The full instrument is available from the authors on request.

The penultimate version of the instrument was pilot tested with three senior decision makers representative of the intended target audience (i.e., vice-presidents of Regional Health Authorities). No substantial revisions were made to the survey instrument or the online administration process in consequence, and results from the pilot test application were pooled with those obtained from the survey roll out for analysis purposes. Completing the survey took respondents an average of 24 minutes (range 15–39 minutes).

Eighty-nine organizations were identified. We sought to obtain three replies per organization, representing different functional roles where possible, for a maximum response of 267 individuals in total. Contact names and email addresses were obtained primarily from publicly accessible websites or posted email contacts. Some organizations (n=4) declined to provide this information and for some we were unable to obtain it during the survey period (n=4). Thus, we were able to approach decision makers from 81 organizations to participate in this survey. In some organizations, fewer than three names were available to contact (due to the small size of the senior executive team, or to pre-selection of possible respondents by the organization). Taking these restrictions into account, the maximum possible response can be adjusted to 244.

Contacts were chosen to represent each of three different roles, where possible: finance, operations, and planning (defined within the survey instrument). Where more than one executive member was available within one of these categories, the contact was chosen randomly. Contacts were invited to participate in the survey by email through clicking on a survey link and entering an individualized password. The survey was hosted on a secure server maintained by the UBC Faculty of Education (EduData). After approximately three weeks and one reminder, initial contacts who did not respond were replaced by a new name; the same procedure was followed through four waves or until contact names were exhausted. The survey period lasted from late-January through late-April, 2011. Respondents were able to reply to either an English or French language version of the survey.

## Results

Four sections follow. The first reports on response rate and information about the respondents. Sections B through D organize findings around the key themes of type of process, assessments of process fairness, and respondents’ overall rating of their priority setting and resource allocation efforts.

### A. Respondents

Ninety-two (92) replies were retained for analysis: 88 complete and 4 substantially complete (at least 80%, or 18 of the main 22 questions answered in full) – 80 in English and 12 in French. We achieved 34.4% of the ideal target (92/267), or 37.7% of the adjusted target (92/244); response rate among all managers contacted across the four waves of data collection (n=410) was 22.4%.

At least one reply was received from every province or territory (see Table 1). Geography is a potential proxy for many organizational features, such as socio-economic or political context, or institutional aspects of the healthcare system that in Canada can vary across jurisdictions due to the provinces’ constitutional authority over healthcare services. However, we do not have enough responses to carry out meaningful sub-analyses on this dimension.

**Table 1:** Geographic distribution of responses

						Provincial/territorial demographics*			
**Region**	**Province/ territory**	**Number of replies**		**Number of organizations represented**		**Population (‘000s)**	**Population per organization (‘000s)**	**Median economic family income ($Can)**	**Health spending per capita ($Can)**
West	British Columbia	12	45	6 of 6	25	4,113	68.6	65,787	5,700
	Alberta	2		1 of 1		3,290	3290.4	76,526	6,754
	Saskatchewan	13		8 of 13		986	74.5	59,998	6,481
	Manitoba	18		10 of 11		1,148	104.4	60,754	6,518
Central	Ontario	10	23	7 of 14	17	12,160	868.6	72,734	5,849
	Quebec	13		10 of 18		7,546	419.2	59,734	5,469
Atlantic	New Brunswick	4	18	2 of 2	12	730	365.0	54,520	6,318
	Nova Scotia	9		6 of 9		913	101.5	57,078	6,497
	Prince Edward Island	1		1 of 1		136	135.4	56,207	6,336
	Newfoundland & Labrador	4		3 of 4		505	126.4	51,791	7,057
North	Northwest Territories	4	6	4 of 8	6	41	5.2	90,865	9,853
	Yukon	1		1 of 1		30	30.4	78,583	8,916
	Nunavut	1		1 of 1		29	29.5	62,592	13,250
TOTAL		92		60 of 89		31,613	355.2	66,343	5,948

*Data from Statistics Canada, 2006 census, and CIHI National Health Expenditures database.

Sixty out of 89 organizations are represented by at least one respondent (see Table 1). Among respondents, slightly over one-half indicated that their organization’s total annual budget was $500 million or less; 30% reported a budget of greater than $1 billion. The majority of respondents indicated that their budgets had increased over the last three years, whereas about one-fifth of respondents indicated a stable trend and a small proportion (8%) indicated that their organization’s budget had contracted. Based on the researchers’ assessment of population and geographic location, we would suggest that 22% of respondents came from primarily urban organizations (average population of just over 800,000), and 32% from primarily rural or remote organizations (average population of slightly less than 55,000); the remainder of cases would be classed as mixed or indeterminate.

Fifty one percent (51%) of respondents self-identified as carrying out an Operational role; 28% were in Planning portfolios and 21% in Finance roles. About 55% of respondents had an educational background in medicine or another health profession. Responsibilities were related to respondents’ educational background: 80% of Operations, 50% of Planning, but none of Finance senior management team members reported doing their major training in medicine or other health professions. As expected, respondents appear to be quite an experienced group with many years of work in senior management roles, largely within the health system. The mean number of years in senior management was 11.8 (standard deviation 7.0), with a mean of 10.8 years specifically in the health sector (standard deviation 6.9). Respondents on average had been employed by their current organization, or any of its direct predecessors, for 10.6 years (standard deviation 8.9).

### B. Type of process

About one-half of respondents indicated that their resource allocation process was best described as what we would label a formal one, while about one-quarter deemed it historical and one-quarter felt that resource allocation processes in their organizations were driven primarily by political factors. (See Table 2 for definitions of the different types, developed by the authors for this study—these were presented to respondents in the form of short scenarios).

**Table 2:** **Use of formal**, **historical and political processes in resource allocation**

		**#**	**%**
Politics (External)	Our spending pattern is almost entirely determined by provincial or federal government requirements and expectations. We have very little real freedom to decide which programs or services will be funded, and to what degree.	20	22.2
Politics (Internal)	The squeaky wheel gets the grease. It seems like additional money goes to those Departments and programs which complain the most loudly, and they are also the best at avoiding any cuts. Their arguments aren’t necessarily always evidence based.	2	2.2
Historical	Each Department and program expects to receive about the same amount as in past years. Much of our money is tied up in things that were historically important services, but the organization is slow to adjust its spending to meet changing needs and times.	22	24.4
Formal	We have a formal process which we use to set priorities and allocate resources. Everyone knows what the rules are, and how and why decisions are made. For the most part, strong evidence is needed to justify all spending decisions.	33	36.7
Formal	Our entire budget is reassessed each year, and all Department and program spending needs to be justified in terms of whether or not it meets the organization’s priorities.	13	14.4

“Please indicate which of the following descriptions you think *most closely* matches how resources are allocated across major programs or portfolios by the senior management team in your organization”.

N=90.

**Figure 1:**
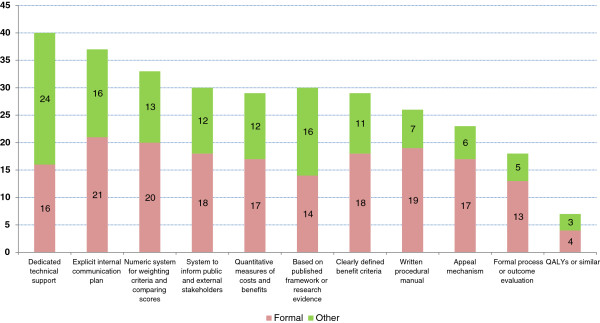
Characteristics of priority setting processes.

We then asked respondents to identify whether or not their organization-wide resource allocation process involved up to 11 elements – identified from the literature – which might be characterized as formalization (See Figure 1). As a group, those who indicated that their organization used primarily a formal process selected on average a larger number of these features: 3.8 compared to 2.8. This suggests that practice within organizations with a ‘formal’ process in fact may differ from that in other organizations. However, it may be that, due to the sequencing of these questions, respondents who stated they used a formal process may have felt compelled to choose more features as justification for this claim.

The above said, none of the features put forward to suggest formalization were reported by even a majority of respondents. The most common feature, dedicated technical support for resource allocation, was reported by 44% of managers. Somewhat surprisingly, respondents from larger organizations reported less use of formal processes; in organizations with budgets greater than $1 billion, 69% of respondents identified historical or political factors as predominant, while in smaller organizations the majority of respondents (59%) cited formal or rational processes as most characteristic. No pattern was observed for role, budget trend, or turnover variables.

### C. Fairness

**Figure 2:**
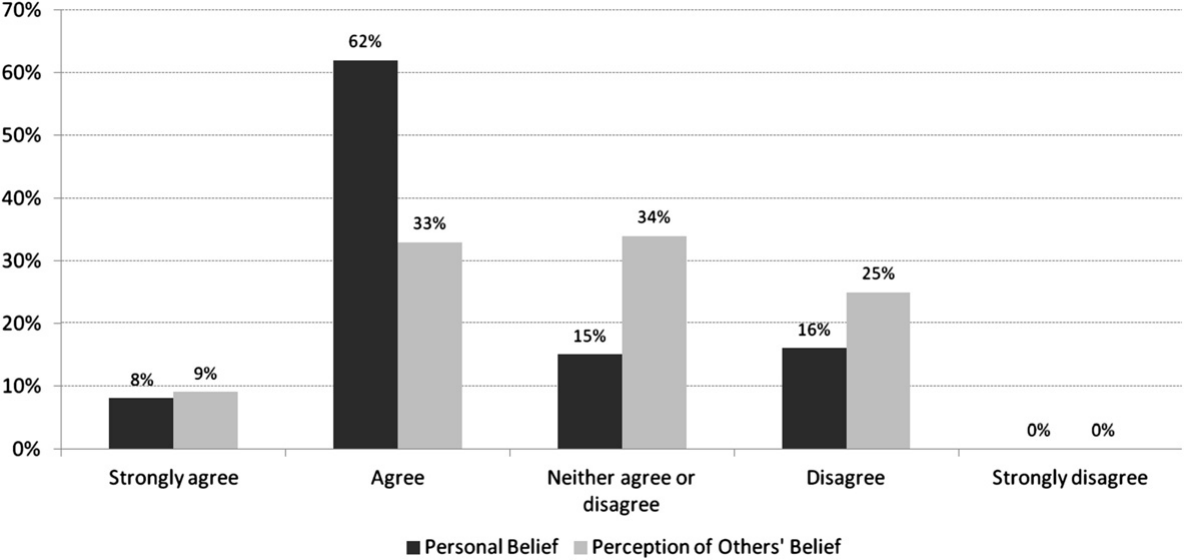
Perceptions of fairness.

Overall, 89% of respondents agreed that resource allocation decisions were not only business ones, i.e., solely financial or budget matters, but matters of ethical concern. 70% of respondents either agreed or strongly agreed with the statement: “Our organization-wide resource allocation process is fair”. Sixteen percent disagreed while 15% were neutral (“neither agree nor disagree”) (see Figure 2). Interestingly, respondents do not appear to think that other stakeholders are equally confident about the fairness of the process. Forty-two percent felt that other key stakeholders would agree that the process is fair, while one-quarter expected that outside observers would consider the organization-wide resource allocation process to be unfair.

As described above, we used the A4R framework to embed questions about concrete resource allocation practices which might be seen as normatively fair. Relevance: Among respondents to this survey, 90% agreed that alignment with vision and mission was a criterion which they used in resource allocation decisions, and 65% said the same about congruence between options and the organization’s corporate values. Publicity: Respondents to the survey were asked if decisions and their rationale were communicated to internal and external audiences. Seventy-seven (77%) agreed or strongly agreed that their organization had a plan to communicate results to internal stakeholders, while 48% gave the same rating in respect to the public and other external stakeholders. Enforcement: One survey item asked whether or not participants felt that “agreed-upon process rules and decisions are followed through on and enforced”. 66% of respondents indicated that this was the case; 23% were neutral and 11% disagreed. However, only 21% of respondents indicated that their organizations evaluated either the process or the outcomes of their resource allocation system. Appeals: Only 24 of 92 participants (26%) stated that there was a formal process by which resource allocation decisions might be reviewed or appealed. Empowerment: Among respondents, one-half (50%) agreed or strongly agreed that “affected stakeholders have a formal opportunity to provide input into the decisions being made”; 24% disagreed.

We also consider some of the organizational and individual factors which might account for respondents’ perceptions of process fairness: primary role and type of process (formal or other). Across each of the three primary roles (finance, operations, or planning), approximately two-thirds of survey respondents ‘agreed’ or ‘strongly agreed’ that their process was fair; i.e., there is no obvious pattern here. By contrast, whether or not respondents agreed or disagreed that their process was fair appears to differ depending on whether or not they indicated that formal processes, or historical or political processes, were predominant (see Table 3).

**Table 3:** **Self**-**reported process fairness**, **and overall rating**, **by type of process reported**

		**Self-report: ‘our process is fair’**			**Self-reported overall process rating**		
		**Disagree**	**Neutral**	**Agree**	**Poor or very poor**	**Neutral**	**Good or very good**
Type of Process	Formal	2%	5%	93%	7%	23%	70%
	Other	28%	26%	47%	18%	50%	32%

### D. Overall rating

Respondents were asked to give an overall rating of their organization-wide resource allocation process, on a scale of 1 to 5, from very poor to very good. Just over 50% rated the process as good or very good, while 37% rated it as fair. Eleven percent (11%) gave a poor rating, while no one stated that the process was ‘very poor’. The proportion of respondents giving good or poor ratings appears to vary depending upon whether they considered a formal process, or historical and political factors, to be the driving force behind organization-wide priority setting (see Table 3). Respondent role and senior leadership turnover showed no obvious pattern. Those from organizations with $1B+ budgets more commonly gave their resource allocation ratings a process of poor or fair than did those with smaller budgets; this seems consistent with the earlier reported results about the effect of organization size.

**Figure 3:**
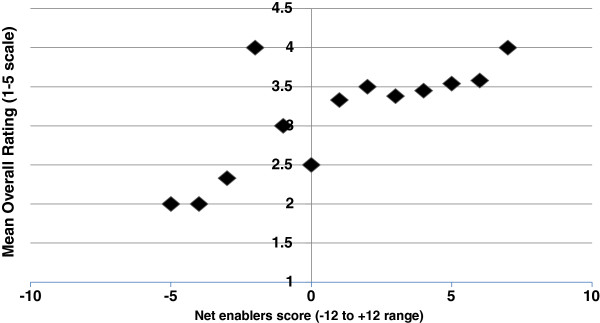
Net enablers against overall rating.

Our survey asked about the presence of a range of potential enablers of effective resource allocation, as identified in the literature, e.g., [29] (See Table 4). Note that most respondents either agreed or strongly agreed that their organizations possessed both a learning culture and strong leadership, though we were unable to explore in depth what these concepts might mean. For each case, we calculated a cumulative or total enabler score: Score +1 for presence of enabler; 0 for “neither agree nor disagree”, -1 for absence of enabler. The range of possible scores is then −12 to +12 (number of individual responses valid on all items=85). Briefly, scores ranged from +7 to −5 (median, +3), with 14% of respondents reporting net negative scores on this measure. It appears strongly suggested by scatterplot data that respondents who identified a greater number of enablers more commonly gave higher overall scores to their current resource allocation procedures (see Figure 3). [The graph groups all respondents by their score, -5 to +7, and then presents the average overall rating given by each group].

**Table 4:** Presence of enablers and barriers to effective resource allocation

		**% (#)**					
	**N=**	**Strongly disagree**	**Disagree**	**Neither agree nor disagree**	**Agree**	**Strongly agree**	Mean, out of 5**
Resource allocation is closely aligned with other key processes, e.g., strategic planning, budgeting	92	2.2 (2)	8.7 (8)	9.8 (9)	54.3 (50)	25.0 (23)	3.82
We have a learning culture	92	1.1 (1)	9.8 (9)	14.1 (13)	58.7 (54)	16.3 (15)	3.79
We have strong leadership, including the presence of a champion for resource allocation processes	92	2.2 (2)	7.6 (7)	16.3 (15)	59.8 (55)	14.1 (13)	3.76
Management personnel have appropriate skills, knowledge, and capacity to implement the resource allocation process as intended	91	2.2 (2)	11.0 (10)	22.0 (20)	54.9 (50)	9.9 (9)	3.59
We have effective process management/facilitation	92	2.2 (2)	9.8 (9)	33.7 (31)	51.1 (47)	3.3 (3)	3.43
‘Politicking’ among participants, unwillingness to engage in ‘honest’ argumentation, efforts to ‘game the system’, [etc.] are [rare]*	92	3.3 (3)	23.9 (22)	27.2 (25)	28.3 (26)	17.4 (16)	3.33
There is […] trust among stakeholders*	92	0.0 (−−-)	22.8 (21)	33.7 (31)	33.7 (31)	9.8 (9)	3.30
The process is […] perceived as fair by affected stakeholders*	89	0.0 (−−-)	24.7 (22)	33.7 (30)	32.6 (29)	9.0 (8)	3.26
There is […] buy-in from key internal stakeholders*	91	0.0 (−−-)	29.7 (27)	31.9 (29)	31.9 (29)	6.6 (6)	3.15
Time and resource commitment required for our resource allocation process are manageable	90	5.6 (5)	26.7 (24)	20.0 (18)	44.4 (40)	3.3 (3)	3.13
We guarantee that no part of the organization will suffer disproportionate losses	92	1.1 (1)	37.0 (34)	38.0 (35)	23.9 (22)	0.0 (−−-)	2.85
We [have] sufficient data to make evidence-informed decisions*	92	13.0 (12)	34.8 (32)	20.7 (19)	27.2 (25)	4.3 (4)	2.75

*=reverse coded.

** One to five scale, where 1=strongly disagree and 5=strongly agree.

## Discussion

Our survey of senior decision makers has provided us with much data on current resource allocation practices within Canadian healthcare organizations. This enables us to investigate critical questions such as in what areas are decision makers performing well, in what areas are there common or shared difficulties, and what factors might be driving successful resource allocation. In our analyses, we focused on three main concepts: type of process, perceived fairness, and overall rating. Only one half of respondents indicated that their organization had a formal process in place. A solid majority suggested that they would consider their own process to be fair, and 51% stated that they would assess their process as ‘very good’ or ‘good’. These measures appear closely linked. The picture is less clear when summary measures are cross-checked against other items, e.g., features of formalization or Accountability for Reasonableness.

### Type of process

Both academic opinion, and many assessments of what decision makers view as the ‘ideal’, support the idea of more formalized resource allocation processes in the health sector [16,20,37-39]. In our survey, about half of respondents claimed to observe formal processes of resource allocation at the senior management level. Most indicated that these were self-developed, not directly derived from models in the literature (Figure 1). This is admirable initiative perhaps, yet suggests the existence of a knowledge translation gap, whether that is due to lack of opportunities for researcher-decision maker interaction, or the failure of academics to ‘push’ or managers to ‘pull’ relevant research [51]. Fewer respondents identified the presence of the elements of what we would consider main features of formalization, as derived from the literature. Perhaps there is social desirability bias—respondents feel that their organizations should make decisions on this basis. Or perhaps decision makers’ understanding of a formal allocation process contains element other than those so far identified in the literature; we could not have anticipated or asked about the presence of such features. Further research is required.

We also need to note what seems like a counter-intuitive relationship between organization size and type of process identified in this data. We anticipated that large organizations would more likely have formalized resource allocation processes; the results do not confirm this. It may be that large organizations are exposed to more diverse opinions and more open political contestation around decisions. Another possible explanation could be that it is easier to obtain consensus among stakeholders in smaller organizations to implement a particular form of resource allocation, including formal processes if desired. On a positive note, we might take this to mean that there are no systemic barriers which prevent organizations of any size from being able to establish practices that enable high performance in resource allocation.

### Fairness

Our respondents recognized the ethical nature of their resource allocation practice; only 11% agreed that they were simply making business decisions. This contrasts with more than 40% in a US survey (from which we derived this question) [52]. It was notable that respondents, taken as a whole, seemed to suspect that outside observers would perceive their organization-wide resource allocation process as less fair than they themselves do. Further data would be required as to what may account for this discrepancy. It might for instance be mediated by views about the effectiveness of organizational communication. Recent research with healthcare organizations in one Canadian province has found a strong link between perceived fairness and perceived transparency [41]. There is some evidence that decision makers may hold different preferences in regard to priority areas for spending than members of the public or healthcare providers [53,54] which might influence what the groups believe to be fair or unfair about the processes currently being used.

The use of A4R elements as survey items provides a way of checking respondent claims about overall fairness against the presence or absence of specific features which are often considered to exemplify fair process. If adherence to A4R principles constitutes fair process (which is an assumption rather than a given), then we should be somewhat cautious in accepting the self-reported claims about fairness made by our respondents. That is, claims about fairness may not be supported based on the extent to which features thought by researchers to facilitate fairness are actually reported as present by these respondents and described above. Some literature has suggested that decision makers are less attached to the importance of formal appeals mechanisms than to other aspects of A4R [55]. Our data appear consistent with this. This leaves interesting questions as to whether it is the attitudes and beliefs of decision makers, or the theory of what makes ethical practice, which needs to change in order to bring about alignment here.

### Overall rating

We seem to have received generally positive responses from our survey participants (assuming that one takes a grading of ‘fair’ to be a positive response). Decision makers no doubt are working hard to do their best, within constraints. But very few nominated themselves as ‘high performers’, indicating awareness of much work yet to do. The findings related to barriers and enablers support the importance of factors previously identified in the priority setting literature as contributing to or restricting effective organizational performance in resource allocation [29].

### Limitations

As with many surveys, it is possible to question the representativeness of the respondents and the generalizability of responses. However the broad range of responses from many different organizations across the country gives us some degree of confidence that we have tapped a diverse set of informants. Response rate is not out of ordinary for an email survey [56]; the large-scale UK surveys noted above had response rates of 23% [26] and 53% [27]. Given that informants are very busy executives who were approached without advance notice, we are not unhappy with the return. We presume (but cannot be sure) that response was provided by the intended informant, and not delegated to someone else.

A further potential limitation is that we certainly have not measured all the relevant variables. The strength and quality of organizational leadership, for one, may account for whether or not organizations choose to implement formal resource allocation processes, the nature of such processes, their fairness, and perceptions among team members of success. We included one question which touched on this (Table 4) -- most respondents reported that their senior management teams displayed strong leadership. However, we know that leadership is a multi-dimensional concept, though we were unable to disaggregate it here. Reeleder et al. identify some of the needed skills as creating relationships, managing networks, building supportive coalitions, and mobilizing support [57]. Dickinson et al. suggest that among these functions “a number .. tend to be weak, or absent, from leadership skills of health care professionals” [58].

In addition, internal consistency in responses from respondents from the same organization is perhaps best described as ‘fair’ (for ordinal questions, inter-rater reliability calculated with Spearman’s rank correlation coefficient averaged 0.580; for nominal questions, Cohen’s kappa averaged 0.274). This is not necessarily surprising, as previous research has noted the members of senior executive teams can have strikingly different perceptions of organizational processes [59]. Role within the organization may lead to different experiences and vantage points to account for this –though we tested it in our study and did not find it to be an explanatory factor. A host of other factors may also be at play. We only included senior managers in this survey. We might well expect senior managers as a group to believe, for instance, in the fairness of processes which they may have helped craft. Those in mid-level management positions may not necessarily perceive organizational resource allocation processes and results in the same terms, but further research is needed to address this.

## Conclusion

Our findings here help to shape up understanding of dimensions of ‘high performance’ which can be more fully investigated both in our own further research, and in the efforts of other scholars and practitioners. A unique contribution to the literature is our assembly of quantitative data about resource allocation practice from to our knowledge the largest yet pan-Canadian set of senior health system managers. Health leaders can look at their own processes in light of what is reported here; it may help them see where they face common challenges – which recur across different social and political geographies -- and where they are seeing significant relative success. This may point to the places where they can teach, where they can learn, and where they may want to reach out to the research community for further knowledge development and support. There is great opportunity for productive mutual engagement to improve resource allocation within Canada’s health system.

## Competing interests

The authors declare that they have no competing interests.

## Authors’ contributions

CM and NS conceived the study; NS took the lead on writing the first draft of the paper; AD, SP, JG, BU, CD and SB all provided input into study design and implementation, and each provided critical comments on drafts of the paper. All authors read and approved the final manuscript.

## Pre-publication history

The pre-publication history for this paper can be accessed here:

http://www.biomedcentral.com/1472-6963/13/247/prepub
